# Constrained evolvability of interferon suppression in an RNA virus

**DOI:** 10.1038/srep24722

**Published:** 2016-04-21

**Authors:** Raquel Garijo, José M. Cuevas, Álvaro Briz, Rafael Sanjuán

**Affiliations:** 1 Instituto Cavanilles de Biodiversidad y Biología Evolutiva, Valencia, Spain; 2 Departament de Genètica, Universitat de València, Valencia, Spain

## Abstract

Innate immunity responses controlled by interferon (IFN) are believed to constitute a major selective pressure shaping viral evolution. Viruses encode a variety of IFN suppressors, but these are often multifunctional proteins that also play essential roles in other steps of the viral infection cycle, possibly limiting their evolvability. Here, we experimentally evolved a vesicular stomatitis virus (VSV) mutant carrying a defect in the matrix protein (M∆51) that abolishes IFN suppression and that has been previously used in the context of oncolytic virotherapy. Serial transfers of this virus in normal, IFN-secreting cells led to a modest recovery of IFN blocking capacity and to weak increases in viral fitness. Full-genome ultra-deep sequencing and phenotypic analysis of population variants revealed that the anti-IFN function of the matrix protein was not restored, and that the Mdelta51 defect was instead compensated by changes in the viral phosphoprotein. We also show that adaptation to IFN-secreting cells can be driven by the selection of fast-growing viruses with no IFN suppression capacity, and that these population variants can be trans-complemented by other, IFN-suppressing variants. Our results thus suggest that virus-virus interactions and alternative strategies of innate immunity evasion can determine the evolution of IFN suppression in a virus.

Pathogen-associated molecular patterns including un-methylated DNA, double-stranded RNA, and specific viral proteins activate signal transduction pathways that lead to the production of type-I interferon (IFN) and trigger the expression of a large number of genes with antiviral effects in the infected and neighboring cells^1^,^2^. Therefore, the ability to suppress IFN-mediated responses is believed to be a major determinant of viral fitness, and viruses have evolved a myriad of IFN-suppressing mechanisms including inhibition of pathogen sensors, interference with signal transducers, or inactivation of downstream antiviral proteins^3^,^4^,^5^. Host innate immunity is central to our understanding of virulence, since disease symptoms are often contributed by immune responses in addition to the direct pathogenic effects associated with viral replication. Previous experimental work with vesicular stomatitis virus (VSV)^6^, foot-and-mouth disease virus^7^ and plant viruses^8^,^9^,^10^ among others has shown that faster viral growth does not necessarily lead to more severe disease symptoms and that, even when such a correlation exists, fast-replicating low-virulence variants can be identified. For instance, VSV point mutations impairing IFN suppression can exhibit fast short-term growth, yet a markedly attenuated phenotype *in vivo*^6^,^11^.

It is widely accepted that, as a result of their extremely high rates of spontaneous mutation, RNA viruses can rapidly adapt to changing selective pressures and that, from a practical point on view, this adaptability has implications for vaccination, drug resistance, and disease emergence^12^,^13^,^14^,^15^,^16^. Importantly, though, genome size constraints have enforced the evolution of multifunctional proteins in many RNA viruses^17^. This can limit the ability to simultaneously optimize different fitness traits, constraining viral evolution^18^,^19^,^20^,^21^. Here, we sought to experimentally analyze the evolution of IFN suppression in an RNA virus and, to achieve this goal we used VSV, a highly IFN-sensitive, prototypic negative-stranded, non-segmented, RNA virus belonging to the family Rhabdoviridae^22^. In contrast to other viruses which deploy several proteins aimed at blocking specific IFN signaling pathways, evasion of IFN-mediated immunity in VSV is thought to depend essentially on rapid completion of the infection cycle and non-specific inhibition of host gene expression. This inhibition is performed by the matrix (M) protein, which induces a general blockade of host transcription and nuclear mRNA export^23^,^24^. However, M is also a structural protein required for virion assembly and budding^25^, and this multifunctional nature may constrain the evolution of IFN suppression in VSV.

Previous work has shown that deletion of the matrix methionine 51 (MΔ51) drastically reduces the IFN suppression capacity of VSV and produces an attenuated phenotype in IFN-competent cells, but not in cells with defects in IFN signaling^26^. Therefore, the MΔ51 mutant provides a good starting point for investigating the evolution of IFN suppression from an experimental approach. We performed 20 serial transfers of VSV-MΔ51 in IFN-competent cells, which should impose a strong selective pressure for the recovery of IFN suppression capacity. However, surprisingly these transfers only led to partial reduction of VSV-mediated IFN induction and to a relatively modest increase in viral fitness in each of three independent evolution lines. Full-length Sanger sequencing and ultra-deep Illumina sequencing revealed no mutations in the M protein associated with IFN-suppression recovery, uncovering a marked inability of the virus to evolve compensatory changes in this protein. In contrast, reproducible sequence changes were found in the viral phosphoprotein (P), which has not been previously implicated in IFN suppression in VSV. In one of the three evolved lines, IFN suppressing variants were present at very low population frequency, and dissection of intra-population variation indicated that fast growers with no IFN suppression capacity were highly abundant. Furthermore, co-infection experiments suggested that non-suppressing variants could benefit from IFN suppressors at high population density by trans-complementation. We propose a model in which the joint effect of these factors leads to a stable polymorphism in which IFN suppressors show low population frequency. Our results illustrate how, despite the extreme simplicity of RNA virus genomes, unexpectedly complex evolutionary outcomes can emerge from population-level virus-virus interactions.

## Results

### Evolution of VSV MΔ51 in IFN-competent cells

**Figure 1:**
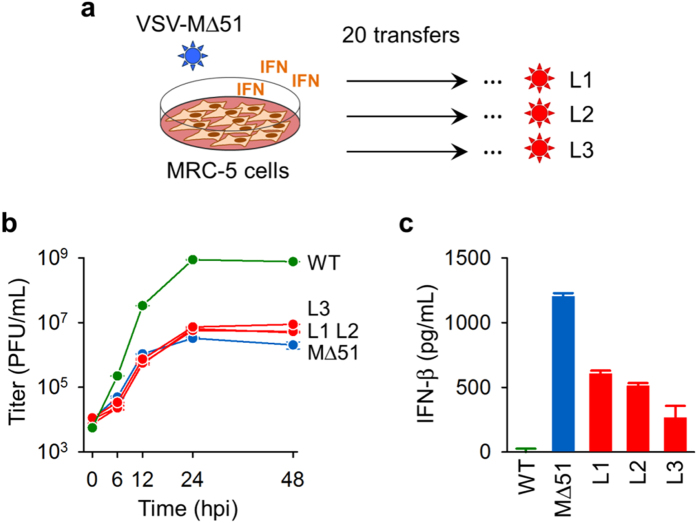
Adaptation of VSV-MΔ51 to IFN-competent MRC-5 cells. (**a**) Scheme of the serial transfers. VSV-MΔ51 was transferred 20 times at an MOI of 0.1 pfu/cell in triplicate (L1–L3). Cells were replaced after each transfer, such that only the virus was allowed to evolve. (**b**) Growth curves of the MΔ51 founder (blue), the evolved lines L1–L3 (red), and a reference virus (WT) showing no defects in the M protein (green). Error bars indicated the standard error of the mean (SEM) from three independent replicates (n = 3). (**c**) IFN-β production in a single infection cycle (MOI = 3 PFU/cell, 16 hpi) as determined by ELISA. Error bars indicate the SEM, with n = 3.

To test the evolutionary stability of the MΔ51 mutant, we performed 20 serial transfers in IFN-competent MRC-5 human lung fibroblasts for each of three replicate evolution lines (L1–L3). The evolved viruses experienced a small increase in fitness as determined by standard growth curves, producing approximately three times more progeny than the founder at 48 hours post-inoculation (hpi), but still two orders of magnitude less progeny than a virus that did not carry the MΔ51 mutation (WT, Fig. 1). Passage-20 viruses showed a two- to fivefold reduction in IFN induction compared with the founder virus, as determined by IFN-β ELISA (MΔ51: 1204 ± 24 pg/mL; L1: 604 ± 10 pg/mL; L2: 511 ± 12 pg/mL; L3:264 ± 92 pg/mL; t-tests against founder: *P* < 0.001; Fig. 1), whereas the WT virus suppressed IFN secretion completely. Therefore, serial transfers led to partial recovery of IFN suppression capacity in the virus and to a significant but small increase in viral growth.

### Restoration of IFN suppression correlates with changes in the viral phosphoprotein

**Figure 2:**
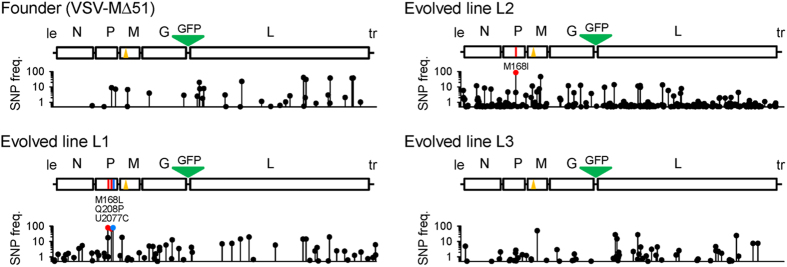
Sequence changes following evolution of VSV-MΔ51 in MRC-5 cells. For each virus (founder, L1–L3), a genome map is shown with the five VSV genes (N, P, M, G, L) indicated in boxes. The leader (le) and trailer (tr) regions were also sequenced by the Sanger method. The MΔ51 virus contained a GFP gene inserted between G and L. Mutations detected by Sanger sequencing are indicated with colored vertical lines (red: non-synonymous, blue: synonymous/non-coding). The original Δ51 deletion is indicated with a yellow triangle and was preserved in all evolved lines. The scatter plots represent the frequency (0.5–100% range) of single-nucleotide polymorphisms as determined by Illumina ultra-deep sequencing. Changes detected by the Sanger method are represented as colored dots. A list of all SNPs detected by Illumina sequencing is provided in Supplementary Data S1.

To identify candidate mutations responsible for these phenotypic changes, we first sequenced the entire genome of the founder and the three evolved lines by the Sanger method, including the leader and trailer regions, which were sequenced after circularization. However, this showed a surprisingly low number of nucleotide substitutions (Fig. 2). The original MΔ51 mutation was preserved in all cases, and there were no apparent secondary changes in the M gene in any of the evolved lines, nor in the nucleoprotein (N), the envelope glycoprotein (G), or the large replicase/transcriptase (L) gene. In contrast, four nucleotide substitutions were found in the phosphoprotein (P) gene. L1 and L2 showed a replacement of the same residue (M168), which was substituted for leucine (CUG) in L1 and for isoleucine (AUA) in L2, whereas L1 had two additional changes (Q208P and a UCU→UCC synonymous change at codon 227). Previous work with VSV^27^,^28^,^29^ and other viruses^30^,^31^,^32^,^33^,^34^,^35^ has shown that parallel sequence changes in replicate evolution lines provide strong evidence for the fixation of selectively advantageous mutations. Therefore, the changes in the M168 residue were probably beneficial, and may be associated with IFN suppression. Illumina sequencing at >5000-fold coverage in >95% of the viral genome revealed that the M168I change was present in 86.0% of the L2 population, whereas the M168L change was present in 78.5% of the L1 population and that, interestingly, the M168I change was also present in 17.5% of the L1 population (Table 1). In contrast, the founder virus exhibited no sequence polymorphisms above the technical detection limit (0.1%) in the vicinity of the phosphoprotein M168 site (±15 residues). As expected for an RNA virus, additional polymorphisms were found in other regions of the viral genome. Of these, 7, 11, 10, and 5 reached population frequencies higher than 10% in the founder, L1, L2, and L3 populations, respectively (Table 1). Most of these polymorphisms were unique to each evolved line and, considering that recombination is very infrequent in VSV^36^, this rules out the possibility that the parallel changes observed resulted from cross contamination. To directly test for the implication of this small region of the P protein in IFN suppression, we engineered the M168I variant in the MΔ51 background by site-directed mutagenesis. Whereas the single mutant MΔ51 produced high IFN levels (1898 ± 137 pg/mL), the double mutant showed full IFN suppression, similar to the WT virus (18 ± 92 pg/mL), therefore showing that the defect introduced in the M protein can be compensated by changes in this residue of the P protein.

### IFN suppressing variants show lower-than-expected population frequency

**Figure 3:**
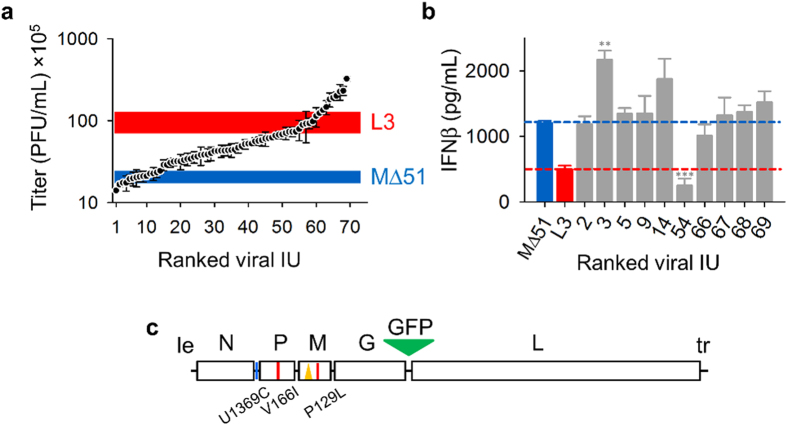
Viral yield and IFN suppression diversity within the L3 population. (**a**) For each of 69 IUs isolated by limiting dilution, the 24 h viral yield (±SEM, n = 3) in MRC-5 cells (MOI = 0.1 PFU/cell at inoculation) was determined. Clones are sorted according to yield rank. The blue and red thick lines indicate the yield (the width of the lines indicates the SEM, with n = 3) of the founder virus and the L3 population under the same assay conditions, respectively. (**b**) Quantitation of IFN-β secretion in a single infection cycle (MOI = 3 PFU/cell, 16 hpi) by ELISA. The red and blue bars and dashed lines indicate the values of the founder and L3 population, respectively. Error bars indicate the SEM (n = 3), and asterisks show the statistical significance of a t-test comparing each IU against the founder (^***^P < 0.001). IU numbers correspond to their viral yield rank in panel a. (**c**) Full-length genome sequence of the c54 isolate. Symbols are as in Fig. 2.

Together, the two substitutions in the M168 residue reached population frequencies of 96.0% and 90.3% in L1 and L2, respectively, indicating quasi-fixation. In contrast, Sanger sequencing of the entire genome of L3 showed no changes, and Illumina sequences confirmed that all polymorphisms in this population were present in <50% of the sequences. Particularly, the M168I substitution was present only in 2.7% of the L3 population, and there was another substitution mapping only two residues away (V166I) which was present in 1.8% of this population. To characterize population variability at the phenotypic level, we isolated 69 individual infectious units (IU) from the L3 population by end-point dilution. The viral yield of these IUs in MRC-5 cells showed a wide distribution, ranging from below the founder virus to above the L3 population level (Fig. 3a). To test whether higher viral yield was associated with stronger IFN suppression, we selected the top four IUs (c66to c69) with highest yields for IFN quantitation. However, none of these IUs recapitulated the IFN suppression capacity of the L3 population and all were indistinguishable from the founder virus in terms of IFN induction, therefore showing a lack of coupling between IFN suppression and replicative capacity (Fig. 3b). In contrast, one IU (c54) with slightly lower viral yield than the other four IUs displayed a 4.8-fold reduction in IFN production (t-test: *P* < 0.001). We also tested five low-yield IUs (c2, c3, c5, c9, c14), but in all cases IFN production was equal to or higher than for the founder virus. Therefore, the IFN suppression phenotype of the L3 line was conferred by minority variants of the population that were not necessarily among the fittest IUs. Full-length Sanger sequencing of the c54 isolate revealed the presence of the V166I substitution in the P protein, reinforcing the conclusion that residues 166–168 are involved in IFN suppression. Two additional changes were found in the c54 genome: a P129L substitution in the M protein and a nucleotide substitution (U1369C) in the spacer region between the N and P genes (Fig. 3c). Illumina data indicated that the P129L change was at 49.3% frequency in the L3 population despite not being detected by Sanger sequencing (Table 1). However, this mutation was not responsible for IFN suppression, because sequencing of this position for the other nine L3-derived IUs indicated that the P129L replacement was also present in c66 and c69, which showed no suppression capacity.

### Lack of association between IFN suppression and growth rate

To address why the ability of the c54 isolate to suppress IFN production may not promote its rapid fixation in the population, we compared the timing of IFN secretion with that of viral growth. In the evolution experiment, each serial infection was initiated at a multiplicity of infection (MOI) of 0.1 plaque forming units (PFU) per cell. We found that MRC-5 cells infected at this MOI did not secrete IFN-β above the limit of detection within the first 10 hpi, regardless of which virus was used. At 6 hpi, the c54 virus produced 2.5 times less progeny that the L3 population (t-test: *P* = 0.016) and approximately the same amount as the founder virus MΔ51 (*P* = 0.219; Table 2). This hence reveals a short-term fitness disadvantage of c54 compared to other members of the L3 population, which may impede its fixation. The MOI increases as the infection progresses and, to reproduce these late conditions, we repeated the above assays using a MOI of 6 PFU/cell. A longer incubation time (16 hpi) was also used in these experiments to allow for putative paracrine effects of IFN secretion that may take place during late-infection conditions. In these assays, cells infected with the founder virus MΔ51 secreted 3.8 times more IFN than those infected with the L3 virus (t-test: *P* < 0.001), and cells infected with the c54 virus showed even lower IFN levels compared to MΔ51 (*P* < 0.001; Table 2). Probably resulting from its increased IFN suppression capacity, the c54 virus produced 4.1 times more progeny than the founder virus under these conditions (*P* < 0.001) and was similar to the L3 population (*P* = 0.448). We then quantified IFN-β in cells co-infected with the founder and c54 viruses at 1:1 input ratio (MOI = 3 pfu/cell each, 16 hpi). Interestingly, IFN secretion was not additive, since the levels observed in co-infected cells were 3.0 times lower than the average for each virus alone (one-sample t-test: *P* = 0.012). Therefore, the IFN suppression activity of the c54 virus was trans-dominant, meaning that it could be exploited by non-suppressing variants of the L3 population in co-infected cells. In contrast, this effect did not result in higher viral titer, since co-infected cells produced slightly (1.5 times) but significantly less total viral progeny than expected under additive effects (one-sample t-test: *P* = 0.001).

### A model explaining the low frequency of IFN suppressors

**Figure 4:**
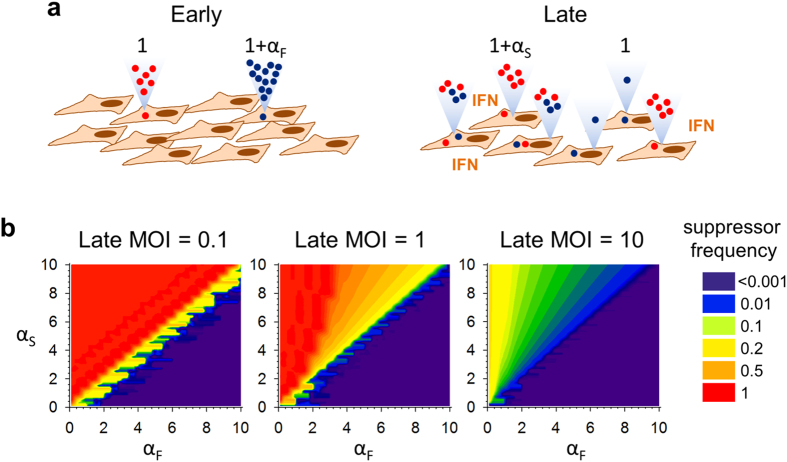
Fitness tradeoffs model for the evolution of IFN suppression. (**a**) Conceptual scheme of the model. Host cells are pictured in brown, and the IFN-suppressing and non-suppressing variants in red and blue, respectively. Released viral progeny is represented with dots inside triangles. During early steps of the infection, the virus spreads rapidly in the absence of IFN, and a fast-growing variant has a relative fitness of 1 + α_F_. During late infection, viral spread is partially inhibited by IFN-mediated antiviral responses, and hence IFN suppressors have a relative fitness of 1 + α_S_. In cells co-infected with fast growers and suppressors, relative fitness differences are offset because viral products are provided in trans. (**b**) Numerical solution of the model for three different MOIs and a range of α_F_ and α_F_ beneficial selection coefficients. Alternated first and second cycles were simulated until a stable equilibrium was reached or one of the two variants became fixed. Colors indicate the final population frequency of the suppressor variant. Q_S_ < 0.0001 (purple) indicates loss of the IFN suppressing virus, and Q_S_ = 1 (red) indicates fixation.

The above data suggest that, in line L3, there may be two coexisting strategies for overcoming IFN-mediated antiviral responses, one consisting of suppressing IFN secretion (suppressors) and another consisting of rapid growth (fast growers), with fast growers dominating the population. Let us assume that fast growers have a fitness advantage α_F_ relative to the founder virus such that per each progeny virus produced by the founder the fast growers produce 1 + α_F_ progeny viruses. In the first infection cycle (within approximately 10 hpi for VSV), before IFN secretion is produced, suppressors do not have any advantage and hence the ratio (R) of fast growers to suppressors after this first cycle is R_1_ = R_0_(1 + α_F_) (Fig. 4a). In the next infection cycle, though, when the MOI becomes high and the IFN-mediated antiviral response has been onset, suppressors benefit from a fitness advantage α_S_, such that R should be now modified by a factor 1/(1 + α_S_). However, fitness differences are offset in cells co-infected with both variants because viral products, including those allowing for IFN suppression, can be provided in trans. As a result, the ratio of fast growers to suppressors after the second cycle equals R_2_ = R_1_/[P + (1 − P)(1 + α_S_)], where P is the probability of co-infection with the two variants. In the model, a stable polymorphic equilibrium is reached when the fitness of fast growers equals that of suppressors, hence if P + (1 − P)(1 + α_S_) =  1 + α_F_. Since 0 < P < 1, a necessary condition for the equilibrium is α_S_ > α_F_, which is a biologically plausible scenario assuming that IFN suppression is an important fitness component. However, P depends in turn on the population frequency of each variant. Assuming that the number of infecting particles per cell is well-described by a Poisson distribution with mean equal to the MOI, the co-infection probability can be expressed as P = [1 − Po(0|Q_S_ × MOI)][1 − Po(0|Q_F_ × MOI)], where Po denotes the Poisson distribution and Q_S_ and Q_F_ the population frequency of suppressors and fast growers, respectively. This expression describes the probability that a cell is infected with at least one suppressor PFU and at least one fast-growing PFU. We solved the model numerically by iterating R_1_, P, and R_2_ for multiple cycles until an equilibrium was reached or one of the variants became effectively fixed, where fixation was defined as min[Q_S_, Q_F_] < 10^–4^, i.e. a frequency on the order of the VSV spontaneous mutation rate^37^. Using a second-cycle (late) MOI ≪ 1 PFU/cell, suppressors reached fixation for α_S_ > α_F_ whereas the fast growers reach fixation for α_S_ < α_F_, and there was no parameter space allowing for a stable polymorphism. However, as the late MOI increases, a stable polymorphism was reached for a broad parameter range, the frequency of IFN suppressors at equilibrium depending on the choice of fitness values for each variant (Fig. 4b). In our experimental setup, first and second cycle conditions were alternated for 20 transfers, with MOI = 0.1 PFU/cell in the first cycle. Assuming a per-cell viral yield on the order of the hundreds^38^,^39^, the MOI was >10 PFU/cell in the second cycle of each transfer, thus allowing for extensive co-infection. Based on the model, for a late MOI of 10 PFU/cell, suppressors should stay as minority variants (<20%) for the vast majority of α_S_ and α_F_ values. By allowing for the presence of non-suppressing, fast-growing variants and trans-complementation, it is thus possible to explain the lack of fixation of IFN suppressing variants using natural selection alone. The model did not include other basic evolutionary processes such as random genetic drift or spontaneous mutation, which may also contribute to the lack of systematic fixation of IFN suppressors.

## Discussion

Previous work with VSV has shown that point mutations with deleterious fitness effects are highly likely to revert to the wild-type sequence after few viral generations^40^,^41^,^42^, but the fact that here the attenuating mutation was a deletion made reversion unlikely, enforcing the selection of secondary, compensatory changes. However, no such mutations were found in the M protein after roughly 40 generations (i.e. 20 transfers with two infection cycles per transfer). This was surprising, because M is the only known IFN suppressor in VSV. In contrast, changes were concentrated in the P protein. Residue 168 of this protein was substituted in 96.0%, 90.3%, and 2.7% of the L1, L2, and L3 populations, respectively, and residue 166 was substituted in 1.8% of the L3 population, as opposed to no detectable (<0.1%) polymorphisms in the founder population. Interestingly, examination of publicly available sequences of the VSV P gene indicates that site 168 is polymorphic, showing the same three residues found here (methionine, isoleucine, and leucine), and that site 166 is also polymorphic (valine and isoleucine). This suggests that this region may act as a modulator of IFN suppression in VSV *in vivo*. The known function of the VSV P protein is to mediate the interaction between the L protein and the viral ribonucleocapsid^43^, and VSV P has not been implicated previously in IFN suppression. Intriguingly, though, in the related rabies virus the homologous phosphoprotein acts as an IFN suppressor by interfering with the phosphorylation of IRF-3 and STAT cellular factors^23^. In VSV, residues 166–168 are in a beta strand located at the end of the central domain of P protein (residues 107–177)^44^. In contrast, STAT binding is dependent on the 10 C-terminal domain residues of the rabies virus phosphoprotein^45^, whereas the interaction with IRF-3 is still poorly characterized. Future work may elucidate whether abolishment of the anti-IFN function of the VSV M protein promotes the de novo evolution of VSV P as an IFN suppressor, or backward evolution to an ancestral state similar to present-day rabies virus.

Changes in the P protein associated with IFN suppression were quasi but not fully fixed in lines L1 and L2, and IFN suppressing variants were present at a considerably low population frequency in the L3 line. The lack of nucleotide substitutions in the entire L3 consensus sequence underscores the importance of analyzing intra-population diversity for understanding the genetic determinants of viral fitness and phenotype. Recent work with Coxsackie virus has also shown that low-frequency population variants play a critical role in the evolution of cell tropism, and suggests that these variants can interact cooperatively^35^. Our data suggest possible factors impeding the fixation of IFN suppressing variants. First, the low-frequency suppressor c54 found within the L3 showed lower viral yield than other, non-suppressing members of the population. Second, this variant was capable of trans-complementing non-suppressing viruses in co-infected cells. A simple deterministic model that incorporates these factors indicates that IFN suppressors can be maintained at low equilibrium frequency, in good agreement with the data. However, it remains unclear why L1 and L2 showed near-fixation of amino acid substitutions in the P protein, whereas L3 did not. We speculate that in the L1 and L2 lines there were variants that combined fast growth and IFN suppression capacity, whereas in L3 these two selectively advantageous traits did not appear in the same variant. The low recombination rate of VSV^36^ makes it unlikely that different beneficial mutations are brought together into the same genome, leading to clonal interference between lineages with different advantageous mutations^46^. As such, historical contingencies related to the order of appearance of mutations may have important effects on the evolutionary outcome, possibly explaining the differences observed between L3 and the other two lines.

VSV-MΔ51 and other mutants with impaired IFN suppression ability such as the L123W/H242R double mutant of the M/G proteins in the related Maraba virus are under preclinical assessment as candidate oncolytic replicating viruses^11^,^26^,^47^,^48^. These mutants are strongly attenuated in normal cells, but they efficiently replicate in cancer cells because the latter often have defects in IFN signaling that allow them to escape immune surveillance, rendering then highly susceptible to viral infections^49^. However, the genetic stability of attenuated viral mutants has not been carefully evaluated^50^. This is a potentially important issue, because reversion to a more virulent, IFN-suppressing form could take place during virus manufacturing or *in vivo* during treatment. Since, in tumors, normal cells are interspersed with cancer cells, restoration of IFN suppression may allow the virus to expand its cell host range and thus could be rapidly selected. Our results indicate that the VSV-MΔ51 mutation is remarkably stable and that no compensatory changes evolve in the M protein after multiple infection cycles in IFN-competent cells. However, this was not accompanied by a similar level of phenotypic stability, since IFN suppression capacity increased after serial transfers. This underscores the importance of evolutionary considerations beyond simple sequencing for evaluating oncolytic virus safety.

## Material and Methods

### Cells and viruses

Primary normal human lung (MRC-5) and immortalized baby hamster kidney (BHK-21) fibroblast were obtained from the American Type Culture Collection (ATCC) and cultured in Dulbeco’s modified Eagle’s Medium (DMEM) (Invitrogen) supplemented with 10% fetal bovine serum (FBS) (Invitrogen). All cells were incubated at 37 °C in a 5% CO_2_ humidified incubator. The VSV-M∆51 mutant has been previously described^26^ and was obtained from the laboratory of Prof. John. C. Bell (Ottawa Hospital Research Institute). This mutant belongs to the Indiana VSV serotype and carries a GFP insert between the G and L genes. We have deposited the sequence of the VSV-M∆51 founder under GenBank accession KU721836.

### Experimental evolution

VSV was subjected to 20 serial transfers (MOI = 0.1 PFU/cell) in MRC-5 cells, in triplicate. Cells were seeded in a 12-well plate at a density of 10^5^ cells/well 24 h prior to inoculation. The inoculum was incubated 1 h at 37 °C to allow for viral adsorption and 1 mL DMEM supplemented with 2% FBS was then added. Supernatants were collected every day at 22–24 hpi, titrated by the standard plaque assay in BHK-21 cells, and used to inoculate fresh MRC-5 cultures.

### Plaque assays

Confluent BHK-21 monolayers were used for titration. After 45 min incubation of the inoculum, cultures were overlaid with DMEM supplemented with 2% FBS and 0.5% (w/v) agarose. After 24 h incubation, cells were fixed with 10% formaldehyde, stained with 2% crystal violet in 10% formaldehyde, and plaques were counted.

### Cytopathogenicity assays

MRC-5 cells were resuspended in DMEM supplemented with 2% FBS at a density of 10^5^ cells/mL in 96-well plates and inoculated at the indicated MOI. At 48 hpi, Alamar Blue (resazurin sodium salt, Sigma) was added at a final concentration of 20 μg/mL and, following a 3 h incubation, fluorescence was quantified with 560 nm excitation and 590 nm emission wavelengths.

### IFN quantitation by ELISA

MRC-5 monolayers were inoculated at a MOI of 3 PFU/cell and, at 16 hpi, 100 μL of the supernatant was collected and incubated in a 96-microtiter plate with standards supplied by the manufacturer (Human IFN alpha ELISA Kit, Pierce). Samples were processed following manufacturer’s instructions and absorbance at 450 nm was quantified.

### Isolation of single infectious units

BHK-21 cells in 96-well plates were inoculated with a dilution of the virus such that approximately 10% of the wells were infected (limiting dilution). Assuming a Poisson distribution of the number of infectious units per well, 95% of the infected cultures should be initiated from a single infectious unit.

### PCR and full-length Sanger sequencing

RNA was extracted from viral supernatants with the QIAAmp Viral RNA minikit (Qiagen). The viral genomic RNA was reverse-transcribed in four fragments using the following sequence specific plus-strand primers: 5′- ACGAAGACAAACAAACCA-3′ (P1, sites 1–18 of GenBank accession KU721836, 5′-GTCTTTTCTATCCCTATG-3′ (P3, sites 3031–3049), 5′-TGGAGATAAATGGCATGAAC-3′ (P5, sites 6905–6925), 5′-GTGGGGACAAGAGATAAAAC-3′ (P7, sites 9590–9610). Reverse transcription was performed from 2 μL purified RNA using AccuScript High Fidelity RT (Agilent), following manufacturer’s recommendations. Each of the four cDNA products was subject to 40 cycles of PCR using the same plus-strand primers and one of the following minus-strand primers: 5′- ACAAGTAGTGACCCATTT-3′ (P2, sites 3321–3339), 5′-TGGGTCTAGTAAGTCGGGTA-3′ (P4, sites 6949–6969), 5′-TATAATGAGCGCCAGTTG-3′ (P6, sites 10519–10537), or 5′-ACGAAGACCACAAAACCAG-3′ (P8, sites 11996–12015). PCR was done with Phusion High-Fidelity DNA Polymerase (Thermoscientific) following manufacturer’s instructions. To amplify the genome ends (leader and trailer), viral supernatants were first concentrated fivefold using Amicon Ultra 15 100 KDa centrifugal filter units (Millipore), and RNA was extracted using TRI Reagent (Sigma). The extracted RNA was circularized using T4 RNA ligase I (New England Biolabs) for 2 h at 37 °C, and reverse-transcribed using AccuScript High Fidelity RT and a plus-strand primer that hybridized near the end of the genome (5′-CACAGGAATGATTGAATGGATCAATAG-3′, sites 11813–11840). PCR (35 cycles) was performed with Phusion High-Fidelity DNA Polymerase, the above plus-strand primer, and a minus-strand primer that hybridized near the start of the genome (5′-GCAGGAAGTTTTGGAACTATGACTGTG-3′, sites 108–135), such that only circularized genomes were amplified. PCR products were loaded in a 2% agarose gel and bands of the expected size were excised and purified with the Zymoclean Gel DNA Recovery Kit. Sequencing was done using the standard Sanger method and chromatograms were analyzed with the Staden package (staden.sourceforge.net).

### Ultra-deep Illumina sequencing

PCR products were fragmented, size-selected, uniquely tagged by adaptor ligation, and sequenced in an Illumina MiSeq machine using pair-end libraries. The quality of the raw sequences was evaluated with FastQC software 0.10.1 (www.bioinformatics.babraham.ac.uk/projects/fastqc). Illumina adaptors and PCR primers were removed with Cutadapt software (code.google.com/p/cutadapt). FastQ files were trimmed and de-replicated using PrinSeq-lite 0.20.4 (prinseq.sourceforge.net). Mapping was done using the Mem algorithm included in Bwa 0.7.12 (github.com/lh3/bwa). SAM files were converted to BAM format, sorted and indexed using SAMtools (samtools.sourceforge.net). VarScan 2.3.7 (varscan.sourceforge.net) was run to call variants using SAMtools mpileup data as input and a minimum variant frequency of 0.1%, a lower-limit imposed by the reading accuracy of this sequencing technology.

### Site-directed mutagenesis

A full length cDNA clone was linearly amplified for 18 cycles using a pair of self-complementary primers carrying the desired mutation and the high-fidelity Phusion DNA polymerase. To remove the template DNA, the product was treated with DpnI, which selectively digests methylated DNA. *E. coli* cells were then transformed by the rubidium chloride heat-shock method and plasmid DNA was purified using the Nucleospin Plasmid purification kit (Macherey-Nagel) and used for transfecting BHK-21 cells as described in previous works^51^,^52^. Briefly, young 90% confluent BHK-21 cells were infected with a recombinant vaccinia virus expressing T7 RNA polymerase, and then co-transfected with the full-length VSV cDNA clone and three helper plasmids encoding the P, L, and N proteins. Transfections were carried out using Lipofectamine LTX (Life Technologies), following manufacturer’s instructions. After 6 h, 25 μg/mL 1-β-D-arabinofuranosylcytosine was added to inhibit vaccinia replication and, after 3–4 days, supernatants were tested for the presence of infectious VSV particles by the standard plaque assay. Vaccinia virus was removed by filtration and one additional blind infection was performed in BHK-21 to increase titer.

### Model

The model equations for numerical simulations were implemented in an R script provided in the Supplementary Note, and plotted using Sigma Plot.

## Additional Information

**How to cite this article**: Garijo, R. *et al.* Constrained evolvability of interferon suppression in an RNA virus. *Sci. Rep.* **6**, 24722; doi: 10.1038/srep24722 (2016).

## Supplementary Material

Supplementary Information

Supplementary Dataset S1

## Figures and Tables

**Table 1:** Genetic diversity of the founder and evolved populations determined by ultra-deep sequencing^a^^,^^b^.

Line	Gene	SNP	Residue	Abundance (%)
Founder	L	G5899A	D105N	19.8
	L	G7365A	–	22.7
	L	A9498G	–	40.6
	L	A9584U	E1333V	30.6
	L	A10420C	I1612L	37.3
	L	C11175U	–	37.0
	L	G11217A	–	37.8
L1	P	A1898C	M168L	78.5
	P	G1900A	M168I	17.5
	P	A2019C	Q208P	65.7
	P	U2077C	–	78.8
	M	C2388A	–	18.6
	G	C4119A	–	12.8
	L	U7326A	–	14.3
	L	C7624U	–	19.6
	L	G9486A	–	14.2
	L	G9591A	–	14.4
	L	C10400A	S1605Y	19.6
L2	N	G543U	–	11.7
	P	G1900A	M168I	86.0
	M	A2472C	R73S	11.4
	M	A2755G	K168E	46.2
	G	A3716U	E210V	11.9
	G	A4138C	I351L	12.6
	L	G6148U	G188C	13.9
	L	G6972A	–	11.2
	L	U7050C	–	12.2
	L	A7820G	E745G	13.5
L3	M	C2639U	P129L	49.3
	L	G6188A	R201K	27.5
	L	A6245G	K220R	14.9
	L	A6963U	–	27.2
	L	U10373A	M1596K	24.1

^a^All SNPs with >10% population frequency are shown except for the marker gene GFP. A full list of SNPs is provided in Supplementary Data S1.

^b^Changes detected by Sanger sequencing are underlined.

**Table 2:** Time-dependency of viral fitness and IFN secretion.

Conditions	Virus	Titer (pfu/mL)	IFN (pg/mL)
Early^1^	MΔ51	(8.7 ± 1.2) × 10^4^	0^3^
	L3	(14.3 ± 2.2) × 10^4^	0
	c54	(6.0 ± 1.5) × 10^4^	0
	MΔ51 + c54	(5.3 ± 0.7) × 10^4^	0
Late^2^	MΔ51	(3.0 ± 0.1) × 10^7^	1068 ± 180
	L3	(11.7 ± 1.1) × 10^7^	283 ± 30
	c54	(11.8 ± 0.3) × 10^7^	76 ± 58
	MΔ51 + c54	(4.8 ± 0.3) × 10^7^	191 ± 42

^1^MOI = 0.1 pfu/mL, 6 hpi.

^2^MOI = 6 pfu/mL, 16 hpi.

^3^Undetectable levels.
