# Supplementary material for: Constrained evolvability of interferon suppression in an RNA virus
Source: Sci Rep. 2016 Apr 21;6:24722. doi: 10.1038/srep24722 (PMC4838867; doi:10.1038/srep24722)
Supplement: Supplementary Information [file srep24722-s1.pdf]

Constrained evolvability of interferon suppression in an RNA virus

Raquel Garijo[1], José M. Cuevas[1], Álvaro Briz[1], Rafael Sanjuán[1,2*]

Supplementary Information

Supplementary Data S1. List of all SNPs found in the founder virus and evolved lines by ultra-deep sequencing (in Excel format).

Supplementary Note. R script of the mathematical model describing the evolution of IFN.

```
IFNmodel <- function(R0,aF,aS,moi,N){

 # This function executes the model described in the paper
 # R0, aF, aS and moi are the variables mentioned in the paper
 # N is the number of cycles performed
 # It is necessary to choose N large enough to allow convergence to the equilibrium value,
 # if it exists

 # The initial frequencies of the two variants can be calculated from R0
 # qS=1/(1+R0), qF=1-qS

 # ratios is a vector that will contain all the calculated ratios
 # the initial ratio is at the first position of this vector
 ratios=rep(0,2*N+1);
 ratios[1]=R0;

 # N cycles are performed
 for (i in c(1:N)) {
  # R1
  R1=R0*(1+aF);
  ratios[2*i]=R1;
  # The frequencies qF and qS are calculated from each R1 that is obtained
  qS=1/(1+R1);
  qF=1-qS;

  # R2
```

```
    # P is a random variable which depends on qF and qS
    P=(1-dpois(0, qS*moi))*(1-dpois(0, qF*moi));
    R2=R1/(P+(1-P)*(1+aS));
    ratios[2*i+1]=R2;
    # R2 is taken as the new R0 and the cycle starts again
    R0=R2;
  }
  # Vector ratios is the output of this function
  return(ratios)
}


IFNmodeltable <- function(vectorR0,vectormoi,x,y,N){

  # This function evaluates the function above, IFNmodel, for a set of values for R0 and moi
  # vectorR0 and vectormoi are the two vectors that contain the values for R0 and moi that we are
  # interested in computing
  # x and y are two partitions of the open interval ]0,1[
  # aS (aF) will take the values contained in x (y)
  # N is the number of cycles that will be performed when using function IFNmodel
  # The output variable, table, will be a matrix with 5 columns (R0, aS, aF, moi and qS) and
  # length(vectorR0) x length(vectormoi) x length(x) x length(y) rows

  # The object table is initialized
  table=c();
  for (R0 in vectorR0) {
    for (moi in vectormoi) {
      for (i in x) {
        aS=i;
        for (j in y) {
          # a new row for the table is initialized
          row=c();
          aF=j;
          #
          ratios=IFNmodel(R0,aF,aS,moi,N);
          # Last element of this vector is extracted (it can be assumed that this is
          # the best approximation to the real equilibrium ratio)
          finalratio=tail(ratios,n=1);
```

```
          # qS is calculated from finalratio
          qS=1/(1+finalratio);
          qF=1-qS;
          # row is completed with the required information
          row=c(row,R0,aS,aF,moi,qS)
          # table is updated with the new row
          table=rbind(table,row);
        }
      }
    }
  }
  colnames(table)=c("R0","aS","aF","moi","qS")
  return(table)

}
```
